# Parenting styles as a moderator in the relation between childhood trauma and psychotic-like experiences among Chinese college students: A cross-sectional study

**DOI:** 10.1097/MD.0000000000047954

**Published:** 2026-03-06

**Authors:** Hongjie Li, Dapeng Wu, Hong Chao, Yuehui Jia, Xueyan Qian, Lei Qi, Siyuan Wan, Gang Li, Baiming Jin, Xiaolei Yang

**Affiliations:** a Department of Preventive Medicine, School of Public Health, Qiqihar Medical University, Qiqihar, Heilongjiang Province, China; b Department of Cardiology, The Second Affiliated Hospital of Qiqihar Medical University, Qiqihar, Heilongjiang Province, China; c Department of Epidemiology and Health Statistics, School of Public Health, Qiqihar Medical University, Qiqihar, Heilongjiang Province, China; d Department of Nutrition and Food Hygiene, School of Public Health, Qiqihar Medical University, Qiqihar, Heilongjiang Province, China; e Department of Environmental Health, School of Public Health, Qiqihar Medical University, Qiqihar, Heilongjiang Province, China.

**Keywords:** childhood trauma, college students, parenting styles, psychotic-like experiences

## Abstract

Childhood trauma (CT)victimization is associated with increased risk of psychotic-like experiences(PLEs) in students. However, little is known about the role of the parenting style in this association. We aimed to investigate the moderating effect of parenting styles in the above association among Chinese college students. To provide help for educators or parents to prevent students from serious psychological and mental diseases caused by CT. We employed a convenience sampling and snowball sampling to investigate the potential moderating effect of parenting style on this association among Chinese college students. A total of 750 freshmen from Qiqihar city, Heilongjiang Province, China, completed the Childhood Trauma Questionnaire, the Prodromal Questionnaire, and the Simplified Parenting Style Scale. The positivity rate of CT and PLEs were 52.5% and 13.6% respectively. The detection rate of CT and PLEs in males all were higher than that in females. This study identified a high correlation between CT and psychotic experiences among college students. Parenting style has a moderating effect on CT-related psychotic experiences. The total moderating effect of 49.97% and the parallel moderating effects of rejection and emotional warmth of parenting styles accounted for 42.47% and 7.5%, respectively. This study highlights the importance of parenting style, suggesting that negative parenting may exacerbate trauma-related PLEs, whereas warm parenting may mitigate trauma-related PLEs.

## 1. Introduction

Psychotic-like experiences (PLEs) encompass delusion-like beliefs and hallucinations that fall below the threshold for psychotic disorders.^[1]^ There is evidence suggesting that individuals who report PLEs have an increased risk of developing clinical psychosis.^[2]^ Symptoms of PLEs include visual and auditory hallucinations, paranoid delusions, bizarre behavior, and beliefs such as someone else controlling one’s body or possessing special powers.^[3]^ Over the past few decades, PLEs have gained significant importance in the field of mental health.^[4,5]^ Numerous studies have consistently shown that PLEs are prevalent in the general population.^[6,7]^ In a recent cross-national analysis involving over 31,000 respondents across 18 countries, the estimated prevalence of PLEs was found to be 7.2%.^[6,8]^ Similar results have been reported in other studies, with estimated prevalence rates of 17% in school-aged children, 7.5% in teenagers,^[9]^ and approximately 5% in adults.^[10]^ Although PLEs do not cause severe pain or functional impairment,^[7]^ their impact can become increasingly severe as life pressures and personal psychological problems escalate. This may lead to difficulties in functioning and living a normal life, and in some cases, individuals may even resort to suicide or engage in antisocial behaviors, which pose significant risks to their physical and mental well-being. Research has demonstrated that experiencing PLEs during adolescence can serve as a predictor of mental disorders in adulthood.^[11]^ Therefore, PLEs are considered predictors of later transition to psychosis and are associated with poor physical and mental health outcomes throughout one’s lifespan.^[12]^ While there is evidence suggesting gender differences in the experience of PLEs, the findings have been inconsistent. Some studies indicate that females report more PLEs.^[13]^ whereas others find a higher prevalence among males.^[14,15]^ Hence, this study aims to further explore the gender differences in the prevalence of PLEs.

Childhood trauma (CT) refers to various types of neglect and abuse experienced by a person during their childhood, including physical, sexual, emotional, and physical neglect, particularly by parents or other caregivers.^[15]^ CT is a significant global public health issue that is closely linked to the mental health of young individuals. It is estimated that over one-third of adults worldwide have experienced CT.^[16]^ Both theoretical and empirical evidence suggests that the prevalence of CT varies greatly across different cultures. For instance, a national sample of American adolescents estimated the prevalence of CT to be 61.8%.^[17]^ While a study conducted among Kenyan and South African adolescents reported higher trauma rates at 83.6%.^[18]^ In China, a survey indicated that approximately 79.01% of adolescents have experienced trauma.^[19]^ CT is a common risk factor for lifelong psychosomatic diseases and plays a crucial role in the pathophysiology of various mental disorders.^[20]^ Furthermore, there may be differences in the incidence of post-traumatic mental illness between individuals of different genders. For example, studies have shown that males who experience trauma have a higher incidence, an earlier age at onset, more negative symptoms, and more cognitive deficits.^[21,22]^ Additionally, previous studies have reported that individuals exposed to CT are more likely to experience PLEs.^[23]^

Parenting style refers to the educational concept and attitude of parents in the process of raising and educating children, as well as their behavior and emotional expression.^[24]^ As the primary caregivers, parents have a significant impact on their children’s emotional health and development.^[25]^ Therefore, parenting style plays a crucial role in determining children’s coping styles, and their behavior towards adults can vary depending on the parenting style. Negative parenting styles can easily lead to psychological problems in children. For instance, an authoritarian parenting style can contribute to hidden emotional problems like depression and anxiety, as well as poor emotional management.^[26]^ Similarly, permissive and uninvolved parenting styles are associated with emotional and behavioral problems in children. On the other hand, an authoritative parenting style aims to create a happy and emotionally stable environment for children, where they feel understood and supported by their parents. Moreover, authoritative parenting helps children face difficulties and adversity better, enhances their resilience to setbacks, reduces negative impacts, and fosters a positive attitude towards life.^[27]^

From the perspective of the stress susceptibility model in developing psychopathology, there is a significant correlation among the 3 factors. Previous studies have shown that individual mental health risks are not determined by a single-factor but result from the dynamic interaction between innate or early-developed susceptibility traits and subsequent exposure to negative stress events. Specifically, CT is a type of negative stressor with high intensity, which directly impairs children’s psychological structure.^[28]^ On the other hand, parenting styles are the core environmental factors that shape children’s vulnerability traits. If parents adopt negative parenting styles such as emotional neglect and harsh punishment, children will gradually develop vulnerability traits like low self-esteem, distrust of the external environment and poor emotion regulation ability.^[29]^ These traits will significantly lower children’s threshold for coping with traumatic stress. when CT occurs, children with high vulnerability are less likely to conduct adaptive cognitive processing of traumatic events, which in turn triggers cognitive distortions, perceptual abnormalities and other PLEs.^[30]^ Conversely, warm, accepting and supportive parenting styles help children build a secure psychological core and sound stress-coping strategies, which weaken the intensity of their vulnerability traits. Even if exposed to CT, children can effectively buffer the negative impacts of trauma, thus reducing the likelihood of developing PLEs.

This study hypothesizes that parenting styles would have a moderating effect on the relationship between CT and PLEs. It aims to analyze the influence of parenting styles on PLEs in college students who have experienced CT. and to provide reference data to help develop targeted psychological education or family interventions to aid in the prevention of PLEs. The study has 3 specific objectives. First, it aims to examine the prevalence of PLEs and CT in Chinese college students, with a focus on gender differences. Secondly, it aims to investigate the associations between parenting styles, CT, and PLEs. Lastly, it aims to determine the moderating roles of different parenting styles in the relationship between CT and PLEs, specifically exploring the indirect contribution of parenting styles to the development of PLEs after experiencing trauma.

## 2. Participants and measurements

### 2.1. Participants

A convenience sampling method was utilized to conduct an online questionnaire survey in March 2023 among freshmen from Qiqihar city, Heilongjiang Provinces, China. Invite respondents to promote the survey questionnaire to eligible individuals in their surrounding area through snowball sampling. Publish survey questionnaire QR codes through WeChat platform, QQ platform, and school mutual aid group. A total of 786 individuals were surveyed, resulting in the collection of 750 valid questionnaires after eliminating 36 invalid ones, achieving an effective rate of 95.41%. Among the valid questionnaires, there were 528 boys and 212 girls.

Sample size estimation: According to a Chinese survey, the detection rate of psychotic experiences among college students is 60.1%.^[31]^ Using the sample size formula: n = *Ζ*^2^_α/2_(1 − *P*)/ε^2^*P*, *P* = .601, with a permissible relative errorεof 10%, α = 0.05, and *Ζ*_α/2_ = 1.96, the calculated minimum required sample size is 255 participants.

This study was approved by the ethical committee of the Qiqihar Medical University (ref: [2025] 60). We carried out all procedures according to the Helsinki Declaration and its subsequent amendments. This survey was conducted in an anonymous form. All participants had signed online informed consent and selected ‘Agree’ at the interviewee’s informed consent. The recruited research subjects should come from Qiqihar Medical College, have normal vision and hearing, no major physical or mental illnesses, and be informed of possible risks and privacy protection regulations.

### 2.2. Measurements

#### 2.2.1. Demographic characteristics

Demographic information collected for the baseline survey included sex, age, hometown, monthly living expenses, history of mental illness, and use of drugs for mental illness.

#### 2.2.2. Measurement of childhood trauma

The Childhood Trauma Questionnaire-Short Form (CTQ-SF) was used to assess traumatic events experienced during childhood.^[32]^ This self-report inventory consists of 28 items. Participants rate each item on a scale of 1 to 5 (1 = never to 5 = always). A positive result in any trauma sub type shall be defined as positive for CT. The specific thresholds are as follows: emotional abuse ≥ 13 points, physical abuse ≥ 10 points, sexual abuse ≥ 8 points, emotional neglect ≥ 15 points, and physical neglect ≥ 10 points. Previous research has established the questionnaire’s reliability and validity in evaluating CT among Chinese college students.^[33]^ In the present study, the total CTQ score demonstrated good internal consistency (Cronbach’s α = 0.874).

#### 2.2.3. Measurement of psychotic-like experiences

The Prodromal Questionnaire (PQ-16)was used to measure symptoms related to the prodromal phase of mental illness.^[34]^ It was a 16-item self-report screening questionnaire, which describes 2 components, the presence of positive and negative symptom items (“true or false”) and the level of distress (calculate by a 4-point scale for each item, from 0 (“no distress”) to 3 (“much distress”). The total score was an addition of all agreed item, higher scores demonstrate adolescents whether were in high-risk status, a psychotic disorder or other psychological disorders. When the item score was ≥7 points or the distress score was ≥8 points, it could be determined as a positive experience of mental illness. It has been confirmed that the questionnaire has good reliability and validity in evaluating the PLEs of Chinese college students.^[35]^ In this study, there were good internal consistency among the total score, item score, and pain score of the scale, respectively (Cronbach’s α = 0.905, 0.898, 0.879).

#### 2.2.4. Measurement of parenting styles

The Egna Minnen av Barndoms Uppfostran (EMBU) was developed to measure parenting styles.^[36]^ However, due to the large number of questions, Arrindell et al selected 46 questions from the standard version of EMBU to create a shorter version called the Short-Egna Minnen av Barndoms Uppfostran (s-EMBU).^[37]^The s-EMBU consisted of 23 items for fathers and mothers, divided into 3 dimensions: emotional warmth, rejection, and overprotection. Each item was rated on a 4-point Likert scale (1 = never, 2 = occasional, 3 = often, 4 = always). When Jiang et al^[38]^ adapted the short-form Egna Minnen av Barndoms Uppfostran for use in Chinese, they found that the items “I feel that my parents prefer my brothers and sisters” and “If I do something wrong, my parents always look sad, making me feel guilty or guilty” were not suitable for Chinese participants and thus were excluded. Therefore, this study utilized a 21-item short-form Egna Minnen av Barndoms Uppfostran in Chinese (s-EMBU-c). The internal consistency of the scale was found to be relatively good in the present study, with Cronbach’s α ranging from 0.80 to 0.89 across all dimensions.

### 2.3. Statistical analysis method

IBM SPSS Statistics for Windows, Version 22.0 (IBM Corp., Chicago) was employed for data analysis. The rate and chi-square test were utilized to examine the detection and differences in past trauma and psychotic experiences. Pearson correlation was employed to explore the correlations among experiences of CT, the PLEs, and parenting styles. The mean ± standard deviation (M ± SD), 2 independent sample *t*-tests, and multiple linear regression analysis were conducted to analyze the prediction of CT on college students’ PLEs. Model 4 from the Process Macro for Windows SPSS was used to construct our moderation models. The bias-corrected percentile bootstrap method with 5000 repeated samples was employed to test the moderating effect, and the level of significance was set at 0.05 (2-sided).

## 3. Results

### 3.1. Common method bias

The data of this study were collected through the self-report method of participants, which may be subject to the risk of common method bias. Although efforts were made to minimize such bias at the research design stage – including anonymous questionnaire filling and varied item wording – to further enhance the rigor and scientificity of the research results, Harman’s single-factor test was adopted to assess the degree of CMB. The results showed that unrotated exploratory factor analysis identified 14 factors with eigenvalues >1, and the first factor explained 28.44% of the total variance. This value was below the 40% threshold, indicating that there is no serious overall common method bias in the data of this study.

### 3.2. Demographic descriptive analysis of psychotic-like experience, childhood trauma and parenting style

In our study, we found no statistically significant difference in the total score of parenting styles based on gender, residency status, living expenses per month, and father’s educational background (*P* = .64, .51, .18, and .09 respectively). Additionally, we observed that overprotection did not show any statistically significant difference between gender and residency status (*P* = .17 and .35 respectively). However, we did find significant variations in the scores of PLEs, emotional neglect, emotional abuse, physical neglect, physical abuse, sexual abuse, CT, rejection parenting style, and emotional warmth parenting style across all demographic variables (*P* < .05). Further details are provided in Table 1.

**Table 1:** Demographic descriptive analysis of psychotic-like experience, childhood trauma and parenting style.

	1	2	3	4	5	6	7	8	9	10	11
Total	3.42 ± 6.25	13.22 ± 5.67	6.37 ± 2.17	9.35 ± 2.40	5.37 ± 1.01	5.20 ± 0.62	39.51 ± 9.30	7.60 ± 2.39	10.02 ± 2.89	21.30 ± 3.85	38.93 ± 5.08
Gender											
Male	2.57 ± 5.42	12.82 ± 5.33	6.21 ± 1.98	8.97 ± 2.15	5.28 ± 0.87	5.16 ± 0.54	38.44 ± 8.34	7.37 ± 2.03	10.12 ± 2.75	21.50 ± 3.48	21.50 ± 3.48
Female	5.69 ± 7.62	14.27 ± 6.39	6.81 ± 2.58	10.36 ± 2.72	5.61 ± 1.29	5.31 ± 0.77	42.35 ± 10.99	8.22 ± 3.07	9.77 ± 3.22	20.79 ± 4.66	20.79 ± 4.66
*t*	−3.36	−2.88	−3.04	−6.57	−3.35	−2.51	−4.62	−3.66	1.37	2.00	0.46
*P*	0.00	0.00	0.00	0.00	0.00	0.01	0.00	0.00	0.17	0.04	0.64
Highest education attained of Father											
university	1.69 ± 4.07	12.34 ± 5.17	5.98 ± 1.76	8.63 ± 2.03	5.23 ± 0.78	5.10 ± 0.40	37.27 ± 7.89	6.87 ± 1.40	10.27 ± 2.70	22.08 ± 2.90	39.22 ± 4.35
high school	5.00 ± 7.43	14.76 ± 5.90	6.53 ± 2.01	10.07 ± 2.32	5.38 ± 1.04	5.26 ± 0.70	42.00 ± 8.99	8.28 ± 2.72	9.87 ± 3.09	20.62 ± 4.11	38.77 ± 5.52
junior high school	7.06 ± 8.14	13.89 ± 6.37	7.50 ± 3.11	10.77 ± 2.74	5.85 ± 1.46	5.48 ± 0.94	43.49 ± 11.79	9.07 ± 3.39	9.40 ± 3.13	19.66 ± 5.35	38.13 ± 6.48
*F*	49.16	13.70	25.91	57.09	18.82	20.78	33.43	59.56	4.77	24.56	2.34
*P*	0.00	0.00	0.00	0.00	0.00	0.00	0.00	0.00	0.00	0.00	0.09
Highest education attained of Mother											
university	39.30 ± 4.14	12.05 ± 5.01	5.88 ± 1.72	8.49 ± 1.92	5.19 ± 0.69	5.08 ± 0.38	36.69 ± 7.51	6.88 ± 1.37	10.28 ± 2.71	22.15 ± 2.75	39.30 ± 4.14
high school	38.69 ± 5.69	14.94 ± 5.90	6.71 ± 2.06	10.19 ± 2.38	5.44 ± 1.16	5.27 ± 0.72	42.55 ± 9.08	8.11 ± 2.66	9.78 ± 3.01	20.80 ± 4.09	38.69 ± 5.69
junior high school	38.11 ± 6.47	14.06 ± 6.41	7.38 ± 3.06	10.73 ± 2.69	5.83 ± 1.41	5.46 ± 0.90	43.45 ± 11.67	9.07 ± 3.41	9.61 ± 3.17	19.43 ± 5.41	38.11 ± 6.47
*F*	48.17	21.14	28.29	71.55	21.00	20.68	46.63	54.00	3.67	28.30	2.98
*P*	0.00	0.00	0.00	0.00	0.00	0.00	0.00	0.00	0.02	0.00	0.05

1 = psychotic-like experiences, 2 = emotional neglect, 3 = motional abuse, 4 = physical neglect, 5 = physical abuse, 6 = sexual abuse, 7 = childhood trauma, 8 = rejection, 9 = over protection, 10 = emotional warmth, 11 = parenting styles.

### 3.3. Detection rates and gender differences in childhood trauma, psychotic-like experiences and parenting styles

More than half (52.5%) of the 750 college students had experienced trauma during childhood, with a higher detection rate in men compared to females (33.9% vs 18.7%, *χ^2^* = 28.10, *P* < .01). The positive detection rate of PLEs among the college students was 13.5%, with a slightly higher rate in males than females (7.1% vs 6.4%, *χ^2^* = 23.96, *P* < .01). Additional information can be found in Table 2.

**Table 2:** Detection rates and gender differences in childhood trauma and psychotic-like experiences.

	Childhood trauma		Psychotic-like experiences		*χ^2^*_*t*_	*χ^2^*_*p*_
	+	-	+	-		
Males	254 (33.9)	291 (38.8)	53 (7.1)	492 (65.6)	28.10**	23.96**
Females	140 (18.7)	65 (8.7)	48 (6.4)	157 (20.9)		
Total	394 (52.5)	356 (47.5)	101 (13.5)	649 (86.5)		

** *P* < .01, *t* is childhood trauma, *p* is psychotic-like experiences.

### 3.4. Correlation analysis between the PLEs and CT and 3 parenting styles

**Figure 1.**
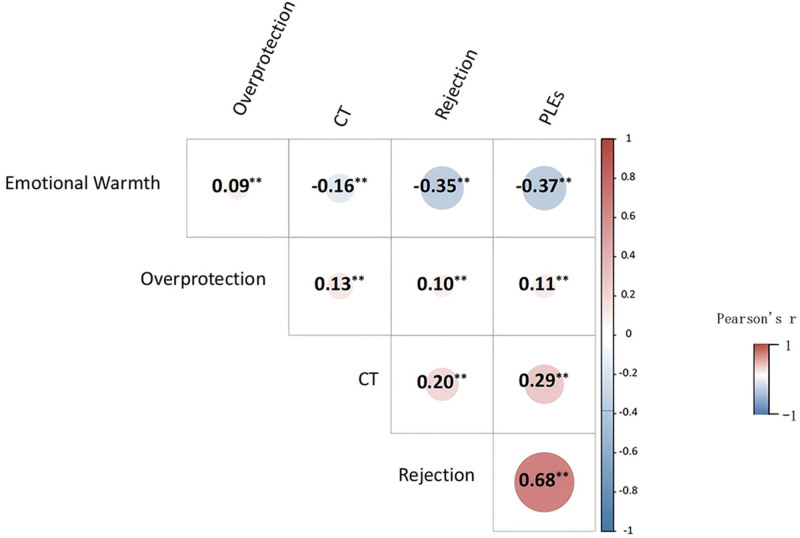
Heat map of the correlation between PLEs, CT, and 3 parenting styles. The redder the color, the stronger the positive correlation; the bluer the color, the stronger the negative correlation. The size and color of the circle represent the strength of the correlation. The larger and darker the square, the stronger the correlation. Values in the circles represent the correlation coefficient *r*. The heatmap shows a significant and positive correlation between CT and PLEs (*P* < .001). Moreover, the study found that parental rearing patterns characterized by rejection and overprotection were significantly and positively correlated with both PLEs and CT (*P* < .001). Conversely, emotional warmth exhibited a significant negative correlation with both PLEs and CT (*P* < .001). CT = childhood trauma, PLE = psychotic-like experiences.

The heatmap shows a significant and positive correlation between CT and PLEs (*P* < .001). Moreover, the study found that parental rearing patterns characterized by rejection and overprotection were significantly and positively correlated with both PLEs and CT (*P* < .001). Conversely, emotional warmth exhibited a significant negative correlation with both PLEs and CT (*P* < .001). Further details are provided in Figure 1.

### 3.5. The predictive effect of CT on PLEs and the moderating effect of parenting Styles

A prerequisite for the moderating effect test was that CT had a predictive effect on PLEs. Therefore, we conducted a hierarchical regression analysis, with PLEs as the dependent variable, and the independent variables had 2 levels: gender, parents’ occupation, and parents’ educational level were included in the first level, whereas CT was included in the second level. The results showed that after controlling for the effects of gender, parents’ occupation, and parents’ educational level, CT still had a positive predictive effect on PLEs (*β* = 0.13, *t* = 5.483, *P* < .001; *R^2^* = 0.17).

**Figure 2.**
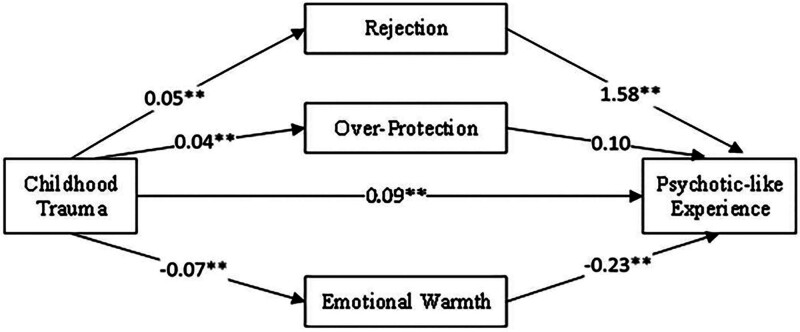
Coefficient of medicine model path. The path diagram illustrated the mediating effect of parenting style on the relationship between childhood trauma and psychotic-like experiences. The value on the arrow indicates the B value of the regression coefficient. Path 1 shown a significant positive relationship between childhood trauma and rejecting parenting style (β = 0.05, *P* < .01), which in turn relates to higher levels of psychotic-like experiences (*β* = 1.58, *P* < .01). Path 2 demonstrated a significant positive relationship between childhood trauma and overprotective parenting style (β = 0.04, *P* < .01), which was associated with a marginal increase in psychotic-like experiences (β = 0.1, *P* = .084 > 0.05). Path 3 revealed a significant negative relationship between childhood trauma and rejecting parenting style (β = −0.07, *P* < .01), leading to a decrease in psychotic-like experiences (β = −0.22, *P* < .01).

The moderating effect of parental rearing patterns on the relationship between CT and PLEs was analyzed using the SPSS macro program. The bias-corrected percentile bootstrap method was employed to test the moderating effect, with 5000 repeated samples. The results, presented in Table 3, indicated that the moderating effect of parenting style was significant (bootstrap 95% CI = [0.066, 0.141]), with an effect value of 0.101. The moderating effects occurred through 2 moderators in parallel. Firstly, the indirect effect of CT→rejection→PLEs (0.083, bootstrap 95% CI = [0.051, 0.119]) indicated a significant moderating effect of the rejection dimension in parenting styles. Secondly, the indirect effect of CT, emotional warmth, and PLEs (0.015, bootstrap 95% CI = [0.005, 0.031]) indicated a significant moderating effect of emotional warmth on parental rearing styles. However, the indirect effect of CT→overprotection→PLEs (0.004, bootstrap 95% CI = [−0.001,0.011]) showed that the moderating effect of the overprotection dimension in parental rearing styles was not significant. The total moderating effect was 49.97%, with the parallel moderating effects of rejection and emotional warmth of parenting styles accounting for 42.47% and 7.5%, respectively (Table 3 and Fig. 2).

**Table 3:** Analysis of intermediary effect.

Indirect effect	Effect value	Boot SE	Boot LLCI	Boot ULCI	Ratio of indirect to total effect (%)
Total	0.101	0.019	0.066	0.141	49.97
Indirect effect 1	0.083	0.017	0.051	0.119	42.47
Indirect effect 2	0.015	0.006	0.005	0.031	7.50
Indirect effect 3	0.004	0.003	−0.001	0.011	

LLCI = lower limit confidence interval, SE = standard error, ULCI = upper limit confidence interval.

### 3.6. Sensitivity analysis

The sensitivity test was conducted by Bootstrap method (5000 sampling), and the results showed that the indirect effect of style between CT and PLEs was 0.093 (standard error = 0.019), the 95% bias-corrected confidence interval was [0.058, 0.129], and it did not contain 0; further included gender as control variables, the indirect effect coefficient 0.082 (standard error = 0.018), CI = [0.047, 0.118], it was still significant the coefficient change range 11.83% < 20%, indicating that the mediating effect has good robustness.

## 4. Discussion

To the best of our knowledge, although previous studies have shown that the relation between different types of parental care, trauma in childhood and psychotic symptoms in adulthood has been proposed,^[39]^ this was the first to explore whether parental rearing patterns have a moderating effect on Childhood Trauma-Associated Psychotic-like Experiences among nonclinical Chinese college students sample. Our findings revealed that 13.5% of the 750 college students experienced PLEs, while 52.5% reported traumatic events during childhood, aligning with a previous study by Wang et al.^[40]^ Wanget al^[40]^ investigated 67,538 students and found that 49.3% of adolescents in China reported at least 1 PLE in the past month. Additionally, Loewy et al.^[20]^ examined risk factors for psychosis and found that a considerable proportion (61%) of clinical-high-risk participants reported trauma exposure. Our survey also identified significant gender differences in the detection rates of CT and PLEs, with males may be showing higher rates than females, consistent with previous research. A trauma survey questionnaire conducted on 16,140 migrant children in China also demonstrated that males had a higher overall risk of trauma exposure compared to females.^[41]^ Males exhibited higher levels of activity and mischievousness compared to females, making them more susceptible to accidents and physical violence.^[42]^ Additionally, males were more likely to witness injuries. One possible explanation for this is that males tend to display insecurity following CT, whereas females are more inclined to seek help, thereby reducing the impact on their sense of security. Furthermore, compared to females, males received more negative parenting styles, possibly due to traditional Chinese values that emphasize the need for males to exercise more authority in discipline.^[43]^ Traumatic experiences are a common occurrence in most people’s lives, and such exposure has been shown to have detrimental effects on both physical and mental health. Moreover, this study discovered that males were more prone to PLEs compared to females, which contradicts previous research findings. The study also identified a strong correlation between CT and PLEs, further supporting the higher prevalence of PLEs in males as compared to females. It is important to emphasize that due to the imbalanced gender ratio in the sample, the observed gender differences in PLEs may not fully reflect the true gender characteristics in the general population. However, supplementary sensitivity analysis confirmed that gender imbalance did not substantially affect the core mediating/moderating effects of the model, suggesting that the association between CT, parental rearing patterns, and PLEs is relatively stable across genders.

Previous studies have indicated a positive association between CT and PLEs, suggesting that adolescents who have experienced trauma are at a higher risk of developing such experiences. A 3-year study also found that adolescents with traumatic experiences are more likely to experience persistent auditory hallucinations.^[19]^ In our study, we also observed a strong correlation between CT and PLEs. Furthermore, we identified the moderating role of parenting style in the relationship between CT and PLEs. Our findings revealed that emotional warmth in parenting style was negatively correlated with CT and PLEs, while refusal parenting style was positively correlated with CT and PLEs. Therefore, We propose that there is an association between exposure to parent-induced traumatic events during childhood and the development of psychotic symptoms, with impairments in cognitive function and emotion regulation potentially acting as factors that influence this associative direction. Parents play a crucial role as the first teachers of their children, influencing their language and behavior. In our study, we examined the moderating effect of parenting styles on the relationship between CT and PLEs. Our findings revealed that parenting styles mediated 49.97% of this relationship. Specifically, parental rejection accounted for 42.47% of the total moderating effects, while parental emotional warmth accounted for 7.50%. It is important to note that high levels of parental rejection and low levels of parental emotional warmth were found to be associated with an increased likelihood of PLEs related to CT among college students. This observation suggests that college students who lack sufficient care and love due to high parental rejection and low parental emotional warmth may be more vulnerable to such PLEs, with these parenting-related factors showing a potential associative direction with PLE vulnerability. Previous research has demonstrated that parenting style is significantly associated with children’s coping styles, and there is variation in children’s behaviors toward adults that correlates with different parenting styles.^[44]^ Consistent with this, among individuals who experienced CT, the absence of parental warm responses or support was found to be associated with a greater tendency to face traumatic experiences alone; this pattern of coping was in turn correlated with feelings of inferiority and resistance in peer interactions or activity engagement.^[45]^ Prolonged exposure to such circumstances was observed to show an association with the emergence of psychological manifestations characterized by delusions and paranoia, known as PLEs.

Consequently, Our study reaffirmed the correlation between CT and PLEs, while also examining the moderating role of parental parenting styles. CT can result in insecure parent-child attachment, negative cognitive and emotional regulation strategies, and a lack of positive strategies, which ultimately contribute to individual psychiatric-like experiences (PLEs). Therefore, trauma therapies and interventions for individuals at high risk of mental disorders should focus on increasing emotional warmth in parenting styles and mitigating the impact of rejecting parenting styles. In terms of intervention measures, parents should adopt a positive attitude towards their children, respond promptly and enthusiastically to their needs and behaviors, respect and encourage their expression of opinions and viewpoints, and provide support and assistance in solving difficulties, especially when their children have experienced trauma. Additionally, interventions such as cognitive therapy or palliative therapy can help alleviate the impact of CT on individuals with psychotic experiences. Creating a highly restorative environment is an ongoing area of research in our study.

### 4.1. Strengths and limitations

This study had several strengths. Although the relationship between CT and the occurrence of PLEs has been previously reported across different countries, our study is the first to be performed in a Chinese population to examine the moderating roles of parenting styles in the link between CT and PLEs. Moreover, the survey subjects were freshmen, reducing information bias.

Despite these strengths, this study had several limitations. First, this was a cross-sectional study, therefore, causal inferences were not avoided. This needs to be further verified by longitudinal studies. Second, the retrospective self-reporting method used to collect the data may have been prone to information bias. Finally, the study participants were chosen from a specific province in northeast China, and representativeness should be considered when interpreting the results. The current study is limited by the imbalanced gender ratio of the participants, which constrains the in-depth interpretation of gender differences in the mediation model. Nevertheless, sensitivity analysis verified that gender imbalance did not substantially alter the core mediation effect between CT, cognitive/emotion regulation, and PLEs. Future research should adopt a stratified sampling strategy with balanced gender representation to further clarify the moderating role of gender in the proposed model and quantify the gender-specific effect sizes of the mediation paths.

## 5. Conclusions

In conclusion, we determined that CT was significantly associated with PLEs and that parenting styles may be related to PLEs through direct or indirect paths via their influence on CT, especially for the dimensions of refusal and emotional warmth. Our major findings highlight the promising role of parenting style in preventing PLEs associated with CT in children and adolescents. This study highlights the importance of parenting, showing that negative parenting can worsen trauma-related PLEs, whereas warm parenting can mitigate trauma-related PLEs. Through increasing warm parenting styles and reducing refusal parenting styles can reduce the PLEs of college students caused by CT. It is recommended that parents utilize a warm and democratic parenting style within the family, particularly by offering positive and meaningful advice and assistance when children face challenges. In addition, our study is among the first to discuss the moderating effect of parenting style on CT and PLEs in the Chinese population. In the future, we will continue to explore other influencing factors, clinical treatment methods, and effective biological diagnostic indicators of PLEs in the Chinese population.

## Acknowledgments

We would like to thank Editage (www.editage.cn) for the English language editing.

## Author contributions

**Conceptualization:** Hongjie Li, Lei Qi.

**Data curation:** Yuehui Jia, Baiming Jin.

**Formal analysis:** Dapeng Wu, Hong Chao.

**Investigation:** Xueyan Qian, Siyuan Wan.

**Funding acquisition:** Hongjie Li, Xiaolei Yang.

**Methodology:** Hongjie Li, Gang Li.

**Project administration:** Hongjie Li.

**Writing – original draft:** Hongjie Li.

**Writing – review & editing:** Xiaolei Yang.

## References

[1] Hinterbuchinger B Mossaheb N. Psychotic-like experiences: a challenge in definition and assessment. Front Psychiatry. 2021;12:582392. 33854445 10.3389/fpsyt.2021.582392 PMC8039445

[2] Lindgren M Numminen L Holm M Therman S Tuulio-Henriksson A. Psychotic-like experiences of young adults in the general population predict mental disorders. Psychiat Res. 2022;312:114543. 10.1016/j.psychres.2022.114543 35417824

[3] Lee KW Chan KW Chang WC Lee EH Hui CL Chen EY. A systematic review on definitions and assessments of psychotic-like experiences. Early Interv Psychiatry. 2016;10:3–16. 10.1111/eip.12228 25772746

[4] Kalman JL Bresnahan M Schulze TG Susser E. Predictors of persisting psychotic like experiences in children and adolescents: a scoping review. Schizophr Res. 2019;209:32–9. 31109737 10.1016/j.schres.2019.05.012

[5] Matheson SL Laurie M Laurens KR. Substance use and psychotic-like experiences in young people: a systematic review and meta-analysis. Psychol Med. 2023;53:305–19. 36377500 10.1017/S0033291722003440 PMC9899577

[6] van Os J Reininghaus U. Psychosis as a transdiagnostic and extended phenotype in the general population. World Psychiatry. 2016;15:118–24. 27265696 10.1002/wps.20310 PMC4911787

[7] Kelleher I Cannon M. Psychotic-like experiences in the general population: characterizing a high-risk group for psychosis. Psychol Med. 2011;41:1–6. 20624328 10.1017/S0033291710001005

[8] Linscott RJ van Os J. An updated and conservative systematic review and meta-analysis of epidemiological evidence on psychotic experiences in children and adults: on the pathway from proneness to persistence to dimensional expression across mental disorders. Psychol Med. 2013;43:1133–49. 22850401 10.1017/S0033291712001626

[9] Kelleher I Connor D Clarke MC Devlin N Harley M Cannon M. Prevalence of psychotic symptoms in childhood and adolescence: a systematic review and meta-analysis of population-based studies. Psychol Med. 2012;42:1857–63. 22225730 10.1017/S0033291711002960

[10] van Os J Linscott RJ Myin-Germeys I Delespaul P Krabbendam L. A systematic review and meta-analysis of the psychosis continuum: evidence for a psychosis proneness–persistence–impairment model of psychotic disorder. Psychol Med. 2008;39:179–95. 18606047 10.1017/S0033291708003814

[11] Isaksson J Angenfelt M Frick MA Olofsdotter S Vadlin S. Psychotic-like experiences from adolescence to adulthood: a longitudinal study. Schizophr Res. 2022;248:1–7. 35907346 10.1016/j.schres.2022.07.010

[12] Healy C Brannigan R Dooley N. Childhood and adolescent psychotic experiences and risk of mental disorder: a systematic review and meta-analysis. Psychol Med. 2019;49:1589–99. 31088578 10.1017/S0033291719000485

[13] Stainton A Chisholm K Woodall T. Gender differences in the experience of psychotic-like experiences and their associated factors: a study of adolescents from the general population. Schizophr Res. 2021;228:410–6. 33556674 10.1016/j.schres.2021.01.008

[14] Laurens K Hodgins S Maughan B Murray R Rutter M Taylor E. Community screening for psychotic-like experiences and other putative antecedents of schizophrenia in children aged 9–12 years. Schizophr Res. 2007;90:130–46. 17207968 10.1016/j.schres.2006.11.006

[15] Stoltenborgh M Bakermans‐Kranenburg MJ Alink LRA van Ijzendoorn MH. The prevalence of child maltreatment across the globe: review of a series of meta‐analyses. Child Abuse Rev. 2014;24:37–50.

[16] Kessler RC McLaughlin KA Green JG. Childhood adversities and adult psychopathology in the WHO World Mental Health Surveys. Br J Psychiatry. 2018;197:378–85. 10.1192/bjp.bp.110.080499 PMC2966503 21037215

[17] McLaughlin KA Koenen KC Hill ED. Trauma exposure and posttraumatic stress disorder in a national sample of adolescents. J Am Acad Child Adolesc Psychiatry. 2013;52:815–30.e14. 23880492 10.1016/j.jaac.2013.05.011 PMC3724231

[18] Seedat S Nyamai C Njenga F Vythilingum B Stein DJ. Trauma exposure and post-traumatic stress symptoms in urban African schools. Br J Psychiatry. 2018;184:169–75. 10.1192/bjp.184.2.169 14754831

[19] Zhang J Liu Z Long Y. Mediating role of impaired wisdom in the relation between childhood trauma and psychotic-like experiences in Chinese college students: a nationwide cross-sectional study. BMC Psychiatry. 2022;22:655. 36271351 10.1186/s12888-022-04270-x PMC9587544

[20] Loewy RL Corey S Amirfathi F. Childhood trauma and clinical high risk for psychosis. Schizophr Res. 2019;205:10–4. 29779964 10.1016/j.schres.2018.05.003 PMC6939986

[21] Kocsis-Bogár K Mészáros V Perczel-Forintos D. Gender differences in the relationship of childhood trauma and the course of illness in schizophrenia. Compr Psychiatry. 2018;82:84–8. 29452966 10.1016/j.comppsych.2018.01.007

[22] Eranti SV MacCabe JH Bundy H Murray RM. Gender difference in age at onset of schizophrenia: a meta-analysis. Psychol Med. 2012;43:155–67. 22564907 10.1017/S003329171200089X

[23] Lu D Wang W Qiu X. The prevalence of confirmed childhood trauma and its’ impact on psychotic-like experiences in a sample of Chinese adolescents. Psychiat Res. 2020;287:112897. 10.1016/j.psychres.2020.112897 32203750

[24] Mak MCK Yin L Li M Cheung RY-h Oon P-T. The relation between parenting stress and child behavior problems: negative parenting styles as mediator. J Child Family Studies. 2020;29:2993–3003.

[25] Ling H Yan Y Feng H Zhu A Zhang J Yuan S. Parenting styles as a moderator of the association between pubertal timing and Chinese adolescents’ drinking behavior. Int J Environ Res Public Health. 2022;19:3340. 35329024 10.3390/ijerph19063340 PMC8954819

[26] Van Der Bruggen CO Stams GJJM Bögels SM. Research review: the relation between child and parent anxiety and parental control: a meta‐analytic review. J Child Psychol Psychiatry. 2008;49:1257–69. 18355216 10.1111/j.1469-7610.2008.01898.x

[27] Garcia OF Fuentes MC Gracia E Serra E Garcia F. Parenting warmth and strictness across three generations: parenting styles and psychosocial adjustment. Int J Environ Res Public Health. 2020;17:7487. 33076230 10.3390/ijerph17207487 PMC7602436

[28] Homberg JR Jagiellowicz J. A neural model of vulnerability and resilience to stress-related disorders linked to differential susceptibility. Mol Psychiatry. 2022;27:514–24. 33649455 10.1038/s41380-021-01047-8

[29] Karcher NR Paul SE Johnson EC. Psychotic-like experiences and polygenic liability in the adolescent brain cognitive development study. Biol Psychiatry Cogn Neurosci Neuroimaging. 2022;7:45–55. 34271214 10.1016/j.bpsc.2021.06.012 PMC8786267

[30] Wang X. Childhood trauma and parenting in at-risk mental state: clarifying pathways and expanding perspectives. World J Psychiatry. 2025;15:112624. 41281542 10.5498/wjp.v15.i11.112624 PMC12635667

[31] Lu D Qing Z Tu Y Liu X. Sexual orientation and psychotic-like experiences among Chinese college students: the role of gender. Front Psychiatry. 2023;14:1139484. 37743983 10.3389/fpsyt.2023.1139484 PMC10514363

[32] Bernstein DP Stein JA Newcomb MD. Development and validation of a brief screening version of the Childhood Trauma Questionnaire. Child Abuse Negl. 2003;27:169–90. 12615092 10.1016/s0145-2134(02)00541-0

[33] He J Zhong X Gao Y Xiong G Yao S. Psychometric properties of the Chinese version of the Childhood Trauma Questionnaire-Short Form (CTQ-SF) among undergraduates and depressive patients. Child Abuse Neglect. 2019;91:102–8. 30856597 10.1016/j.chiabu.2019.03.009

[34] Ising HK Veling W Loewy RL. The Validity of the 16-item version of the Prodromal Questionnaire (PQ-16) to screen for ultra high risk of developing psychosis in the general help-seeking population. Schizophr Bull. 2012;38:1288–96. 22516147 10.1093/schbul/sbs068 PMC3713086

[35] Su N Wang L Shi JY. Reliability and validity testing of the prodromal phase questionnaire (PQ-16) for assessing the risk of mental illness among college students. J Tongji Univ (Medical Edition). 2015;36:123–27.

[36] Perris C Jacobsson L Linndström H von Knorring L Perris H. Development of a new inventory for assessing memories of parental rearing behaviour. Acta Psychiatr Scand. 2007;61:265–74. 10.1111/j.1600-0447.1980.tb00581.x 7446184

[37] Arrindell WA Sanavio E Aguilar G. The development of a short form of the EMBU 1Swedish acronym for Egna Minnen Beträffande Uppfostran (“My memories of upbringing”). 1: its appraisal with students in Greece, Guatemala, Hungary and Italy. Pers Individ Dif. 1999;27:613–28.

[38] Jiang J Zhengrong L Bijing J X Y. Revision of the short-form egna minnen av barndoms uppfostran for Chinese. Psychol Dev Educ. 2010;26:94–6.

[39] Catalan A Angosto V Díaz A. Relation between psychotic symptoms, parental care and childhood trauma in severe mental disorders. Psychiatry Res. 2017;251:78–84. 28189941 10.1016/j.psychres.2017.02.017

[40] Wang D Chen H Chen Z. Current psychotic-like experiences among adolescents in China: identifying risk and protective factors. Schizophr Res. 2022;244:111–7. 35661996 10.1016/j.schres.2022.05.024

[41] Liang Y Zhou Y Liu Z. Traumatic experiences and posttraumatic stress disorder among Chinese rural-to-urban migrant children. J Affect Disord. 2019;257:123–9. 31301612 10.1016/j.jad.2019.07.024

[42] Vakili M Mirzadeh M Mirzaei M. Sex differences in high-risk health behaviors among school-going adolescents in Yazd, Iran; a cross-sectional study. Heliyon. 2023;9:e16404. 37303572 10.1016/j.heliyon.2023.e16404 PMC10250580

[43] Alcaide M Garcia OF Chen F Garcia F. Raising generation Z children in China: parenting styles and psychosocial adjustment. Psychosoc Interv. 2025;34:103–15. 40385638 10.5093/pi2025a9 PMC12082050

[44] Xu C Yan W. Negative parenting styles and social adjustment of university students: a moderated chain mediation model. Curr Psychol (New Brunswick, N.J.). 2022;42:1–14. 10.1007/s12144-022-03809-1 PMC9628611 36340895

[45] Tan R Yang Y Huang T Lin X Gao H. Parent-child attachment and mental health in young adolescents: a moderated mediation analysis. Front Psychol. 2023;14:1298485. 38187411 10.3389/fpsyg.2023.1298485 PMC10768540

